# Development of Efficient Sodium Alginate/Polysuccinimide-Based Hydrogels as Biodegradable Acetaminophen Delivery Systems

**DOI:** 10.3390/gels9120980

**Published:** 2023-12-14

**Authors:** Long Toan Trinh, Saebin Lim, Hyun Jong Lee, Il Tae Kim

**Affiliations:** Department of Chemical and Biological Engineering, Gachon University, Seongnam-si 13120, Republic of Korea; longtt.hust@gmail.com (L.T.T.); binivini1999@gmail.com (S.L.)

**Keywords:** acetaminophen, bioavailability, drug delivery, polysuccinimide, prolonged, sodium alginate

## Abstract

Efficient drug delivery systems are essential for improving patient outcomes. Acetaminophen (AP), which is a kind of oral administration, is a commonly used pain reliever and fever reducer. However, oral administration carries various health risks, especially overdose and frequent use; for instance, AP is administered approximately 4 times per day. Therefore, the aim of this study is to develop an efficient delivery system for once-daily administration by combining sodium alginate and polysuccinimide (PSI) hydrogels to delay the release of analgesic AP. PSI is a biodegradable polymer that can be used safely and effectively in drug delivery systems because it is eliminated by hydrolysis in the intestine. The use of PSI also improves the mechanical properties of hydrogels and prolongs drug release. In this study, hydrogel characterizations such as mechanical properties, drug dissolution ability, and biodegradability were measured to evaluate the hydrolysis of PSI in the intestine. Based on the results, hydrogels could be designed to improve the structural mechanical properties and to allow the drug to be completely dissolved, and eliminated from the body through PSI hydrolysis in the intestines. In addition, the release profiles of AP in the hydrogels were evaluated, and the hydrogels provided continuous release of AP for 24 h. Our research suggests that sodium alginate/PSI hydrogels can potentially serve as biodegradable delivery systems for AP. These findings may have significant implications for developing efficient drug delivery systems for other classes of drugs.

## 1. Introduction

Acetaminophen (AP), a commonly used analgesic and antipyretic, is a component of several prescribed and over-the-counter medicines [1]. However, use of an inappropriate dosage or continuous use of the drug may lead to patient overdose. The recommended dosage of this drug is 500–1000 mg every 4–6 h, with a maximum of 4000 mg every 24 h for both adults and children (≥12 years) [2]. Excess AP can cause acute liver damage. Moreover, AP is one of the most commonly used medicines causing acute liver injury and is the leading cause of acute liver failure as its elimination half-life is approximately 2 h for therapeutic doses [3,4,5,6]. Regular use of AP is essential for analgesic and antipyretic support in patients with fever and discomfort. Although AP is considered a relatively safe drug in the human gastroduodenum, current epidemiological studies have shown that large doses of this drug may increase the risk of bleeding and gastric ulcers [7]. AP is also known to be well absorbed in a wide range of intestinal locations [8]. Therefore, an efficient method for AP delivery to the intestinal tract that retains its analgesic and antipyretic effects for a long period is necessary to reduce its continuous intake and prevent the harmful effects of AP on the body. Nowadays, hydrogels are an interesting class of targeted delivery systems and have been used in numerous biomaterials, including drug delivery systems [9].

Hydrogels are three-dimensional polymeric networks that are hydrophilic, insoluble, and capable of retaining significant volumes of water and biological fluids. They can be used for various biomedical applications, such as the sustained release of therapeutic agents, owing to their biocompatibility and biodegradability [10,11]. Hydrogels have been studied for various biomedical, industrial, and agricultural applications. In the medical field, hydrogels have also been used to enhance recovery and lessen scarring. Hydrogels are excellent materials for injury healing since they have been proven to improve the mechanical and functional properties of animal tissues [12]. They can absorb and store various drugs and facilitate their release at an appropriate rate over time. They exhibit low toxicity and high biocompatibility and biodegradability. Hydrogels can also be completely broken down into monomers or small molecules [13], making them suitable as drug delivery systems to promote sustained and regulated local drug release, improve the therapeutic efficacy of drugs, and decrease the adverse side effects of drugs [14]. Multiple delivery systems based on polymers, such as alginate, are currently being investigated for intestine-targeted delivery of drugs. Digestive system enzymes degrade and release drugs [15,16,17].

Sodium alginate (NaAlg), a natural polymer derived from brown seaweed, is non-toxic and biodegradable. NaAlg is composed of various quantities of d-mannuronic acid and L-guluronic acid. NaAlg has been used for controlled drug release as it rapidly creates three-dimensional networks in the presence of multivalent metal cations [18]. The sodium salt of alginic acid, composed of d-mannuronic acid and L-guluronic acid, chemically creates a hydrogel with the addition of crosslinkers, such as calcium chloride, via interactions with a divalent cation [19]. Under acidic conditions, NaAlg transforms into porous alginic acid, which becomes a soluble layer in the colon [20]. The prolonged release of AP from NaAlg-based controlled-release tablets, as investigated by Rubio and Ghaly, has been the subject of numerous studies on the use of alginate gels for sustained drug release [21]. Sharma et al. tested an in situ quick-gelling formulation for an oral sustained alginate gel for patients with dysphagia [22]. Furthermore, incorporating organic and inorganic elements into biopolymer matrices for reinforcement often improves the mechanical and physical properties of the matrix, which can increase drug encapsulation [23]. Recently, other bioinorganic components, including montmorillonite and CaCO_3_ [24,25,26], have been applied to alginate matrices to generate various alginate–inorganic composite particles that support long-term drug release. Therefore, NaAlg is a suitable drug delivery system.

Polysuccinimide (PSI) is a non-toxic aspartic acid (ASP) polycondensation product [27]. PSI is biodegradable and pH responsive [28]. It is stable under acidic pH and is rapidly soluble at neutral pH due to the hydrolysis of PSI in the succinimidyl primary sequence. This is comparable to the gastrointestinal environment, which has an acidic pH in the stomach and neutral pH in the intestine [29]. A study on the use of PSI in conjunction with AgNPs to boost antibacterial activity and extend the paracetamol release period yielded positive results [30]. PSI nanoparticles (NPs) were shown to hydrolyze slowly under physiological pH 7.4 conditions while being stable under acidic conditions. The ability of NPs to be dissolved under neutral conditions while maintaining stability in acidic environments is a highly desirable property for oral delivery technologies [31]. Therefore, it is considered that PSI exhibits potential for the development of medication delivery systems. The control of drug carrier material degradation in the acidic environment of the stomach using the combination of PSI and NaAlg may aid in prolonging the drug release time in the gastrointestinal environment. PSI is mainly hydrolyzed by the intestinal environment. Therefore, its release in the intestine is important for efficient drug delivery [32]. Furthermore, it is well known that substances that are biologically persistent or that degrade too slowly can cause undesirable biological reactions in the long term [33]. As a result, it is also important to design a drug delivery system that can break it down and release it in the body in a timely manner. The aforementioned qualities and properties of PSI make it a candidate for ensuring the ability of a drug delivery system to hydrolyze substances to extend the drug release time and prevent drug carrier substances from remaining in the body for a long period of time. In this study, we developed an efficient hydrogel system using NaAlg substrate combined with AP-containing PSI to prolong AP release while minimizing drug release in the stomach and maximizing drug release in the intestine. The NaAlg substrate was combined with PSI and crosslinked with Ca^2+^ to form a hydrogel. The biocompatibility and prolonged AP release efficiency of this hydrogel were further studied and compared with those of other hydrogels prepared using only a NaAlg substrate.

## 2. Results and Discussion

### 2.1. Synthesis and Characterization of Alg-PSI

**Figure 1:**
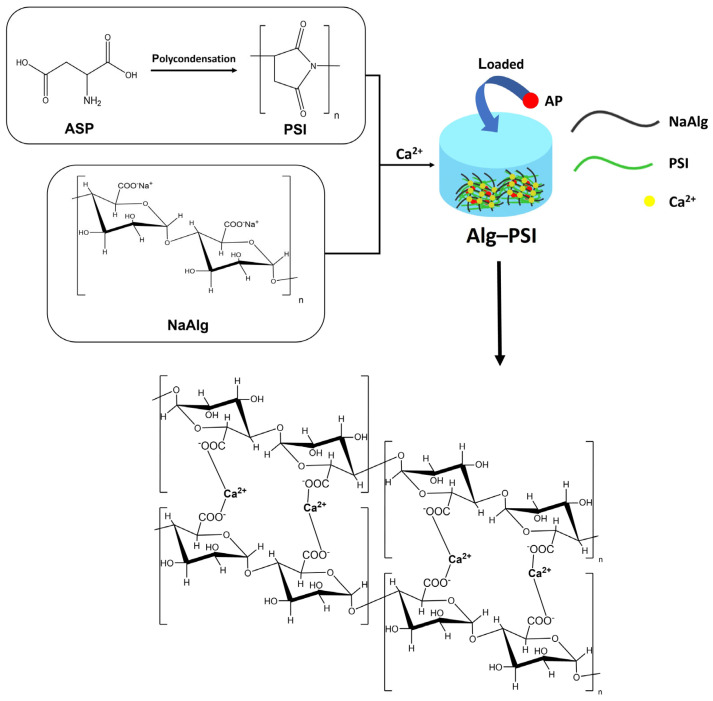
Scheme of hydrogel synthesis using PSI and Alg.

The hydrogel synthesis process comprises two steps, as shown in Figure 1. In the first step, PSI was synthesized by the bulk polycondensation of ASP with an o-phosphoric acid catalyst. ^1^H NMR spectroscopy was used to confirm PSI production (Figure S1). The methine proton signal (a) at 5.3 ppm and the methylene proton signal (b) at 2.7 and 3.2 ppm are visible [34]. The molecular weight of PSI was estimated using gel permeation chromatography (Figure S2). The number average molecular weight (M_n_) and weight average molecular weight (M_w_) were 20,867 and 34,724 g mol^−1^, respectively, with a PDI of 1.66.

**Figure 2:**
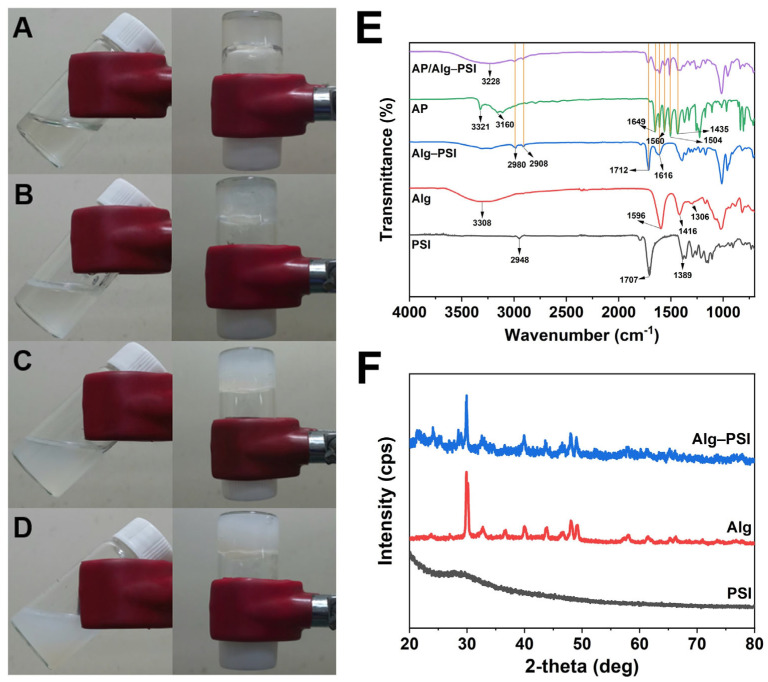
(**A**–**D**) Formation of Alg-PSI hydrogels with different concentrations of PSI: (**A**) 0%, (**B**) 0.5%, (**C**) 1.5%, and (**D**) 3% PSI (*w*/*v*). (**E**) Fourier-transform infrared (FT-IR) spectra of PSI, Alg, Alg-PSI, AP, and AP/Alg-PSI. (**F**) X-ray diffraction (XRD) patterns of PSI, Alg, and Alg-PSI.

The second step involved the synthesis of the Alg-PSI hydrogel. The color of NaAlg and PSI precursor solutions changed markedly when dissolved together (Figure 2A–D). The higher the PSI concentration, the more opaque the NaAlg-PSI mixture. Hydrogel formation was detected using the tube inversion method (Figure 2A–D). The vials were inverted and the flowability of the solution was determined. Gel formation was observed immediately after adding CaCl_2_ solution to the mixture (Figure 2A–D).

FT-IR spectroscopy was used to examine the functional groups of PSI, Alg, and Alg-PSI samples (Figure 2E). For the PSI sample, characteristic bands of the C=O and C-N groups of the imide ring were observed at 1707 and 1389 cm^−1^, respectively [35]. The peak at 2948 cm^−1^ represents -CH_2_ [36]. For the Alg sample, the peaks at 3308, 1596, 1416, and 1306 cm^−1^ represent O-H, -COO- asymmetric, and C-O stretching, respectively [18]. For the Alg-PSI sample, the characteristic peak of Alg was shifted from 1596 to 1616 cm^−1^ and the peak of PSI from 1707 to 1712 cm^−1^, illustrating the combination between Alg and PSI. In addition, the spectra exhibited obvious changes in the characteristic peaks. New peaks at 2980 and 2908 cm^−1^ indicate the presence of the -CH_2_. As a result, the incorporation of PSI into the alginate hydrogel structure is visible. It is noted that when looking into the FT-IR spectrum of Alg-PSI, all characteristic peaks from NaAlg and PSI are still observed even though they show some shifts in peak positions. This may illustrate that there are not any additional chemical reactions between Alg and PSI, but the physical properties have been changed due to the coexistence of Alg and PSI. Meanwhile, the vibrational peaks for O-H and CH_3_ stretching were detected at 3321 cm^−1^ and 3160 cm^−1^, respectively, in the FT-IR spectrum of AP. The vibrational peak at 1649 cm^−1^ was attributed to C=O. At 1560 cm^−1^, the N–H amide II bending was observed. The C–C stretching peak came at 1435 cm^−1^, while the asymmetrical bending in the C–H bond appeared at 1504 cm^−1^ [37]. The AP spectrum reveals an exceptional absorption at around 3321 cm^−1^, which is associated with O–H and not visible in the AP/Alg-PSI spectrum. Rather, a wide peak at 3228 cm^−1^ can be observed. Furthermore, the distinctive peaks for both AP and the Alg-PSI hydrogel are still visible in the AP/Alg-PSI sample, demonstrating that AP and the hydrogel do not react chemically to form byproducts or deteriorate the drug, ensuring the medicinal benefits of AP. It shows that AP was successfully loaded into the hydrogel matrix.

Differences in the structures of PSI, Alg, and Alg-PSI were determined using their X-ray diffraction (XRD) patterns, as shown in Figure 2F. No peaks for PSI are visible in the XRD pattern because PSI is an amorphous polymer [38]. XRD patterns of the Alg and Alg-PSI samples exhibited no significant differences. However, the Alg-PSI sample showed more noise patterns than Alg, possibly due to the incorporation of amorphous PSI.

**Figure 3:**
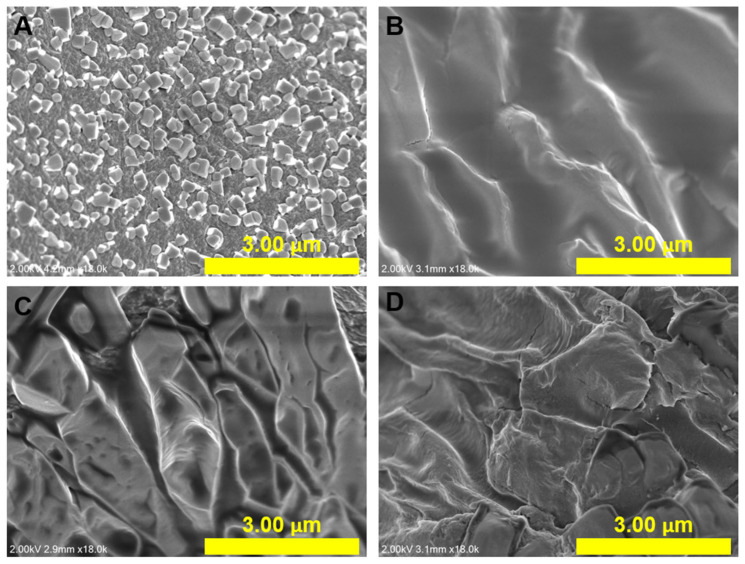
Scanning electron microscopy (SEM) images of hydrogels containing different concentrations of PSI: (**A**) 0%, (**B**) 0.5%, (**C**) 1.5%, and (**D**) 3% PSI (*w*/*v*).

Figure 3 shows SEM images of freeze-dried Alg and Alg-PSI at different PSI concentrations. The surface of the Alg sample showed a similar morphology compared to previous studies (Figure 3A) [39,40]. The Alg surface is rough and contains nanometer-sized particles due to water separation occurring during the freeze-drying process. This causes the polymer networks to break down, forming a rough surface [41]. Here, the Alg-PSI hydrogels had rougher surfaces than those of Alg as PSI was blended into Alg. Surface roughness became more complex as the applied amount of PSI increased from 0.5 to 3% PSI (*w/v*). This can be explained by the insolubility of PSI in water. When PSI is exposed to water, its structure collapses instantaneously, and polymer precipitation is evenly dispersed and blended in NaAlg before this mixture forms a hydrogel with CaCl_2_ crosslinking, leading to a rough surface [31]. This demonstrates that PSI was incorporated into the hydrogel.

### 2.2. Rheological Properties of Hydrogels

**Figure 4:**
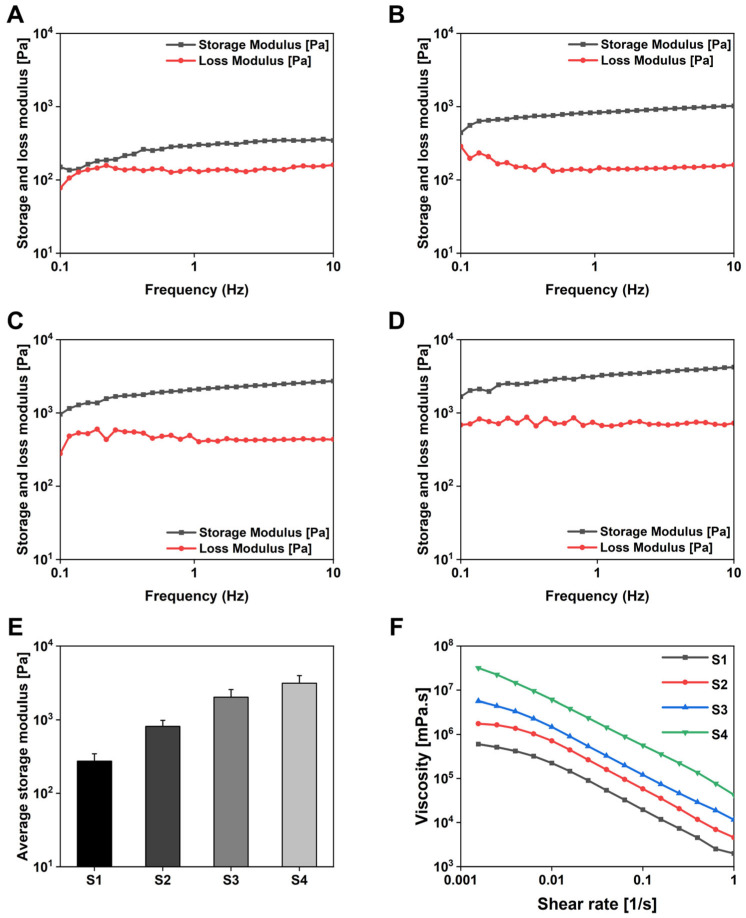
(**A**–**D**) Frequency sweep of dynamic moduli (G’: storage moduli; G″: loss moduli) of Alg-PSI hydrogels at 37 °C. Frequency = 0.1–10 Hz, strain = 0.1%. (**A**) 0%, (**B**) 0.5%, (**C**) 1.5%, and (**D**) 3% PSI (*w/v*). (**E**) Average G’ values of Alg-PSI hydrogels: (S1) 0%, (S2) 0.5%, (S3) 1.5%, and (S4) 3% PSI (*w/v*). Significant differences were observed between all of the groups (*p* < 0.01). (**F**) Viscosity curves of Alg-PSI hydrogels: (S1) 0%, (S2) 0.5%, (S3) 1.5%, and (S4) 3% PSI (*w/v*).

Next, the mechanical properties, stability, and viscosity of the hydrogels were investigated using a rheological test method. Gelation occurred when Ca^2+^ cations were added to Alg, forming an Alg-PSI complex. After 24 h of rest and stabilization, the rheological properties of the hydrogels were analyzed based on the rheological characteristics of the material and frequency variation. Figure 4A–D shows the storage modulus (G’) and loss modulus (G″) of the hydrogels as functions of frequency. G’ was greater than G″ in all samples, indicating that Ca^2+^ cations replaced Na^+^ ion sites to form a stable hydrogel network. The average value of G’ of the hydrogels is calculated in Figure 4E. G’ increased with increasing PSI concentrations in the complex. Moreover, the combination of PSI with Alg made the hydrogel structure more solid and stable, increasing its application stability as a drug delivery system. Figure 4F shows the viscosity of the hydrogels. Viscosity decreased with increasing cutting speed and increased with increasing PSI concentration in the hydrogel network. PSI acted as a thickener in the hydrogel system. PSI may have contributed to the increase in viscosity by improving the interactions with Alg in the hydrogel matrix and increasing hydrogel entanglement.

### 2.3. Drug Loading Efficiency and Degradation Tests to Assess Drug Release

**Figure 5:**
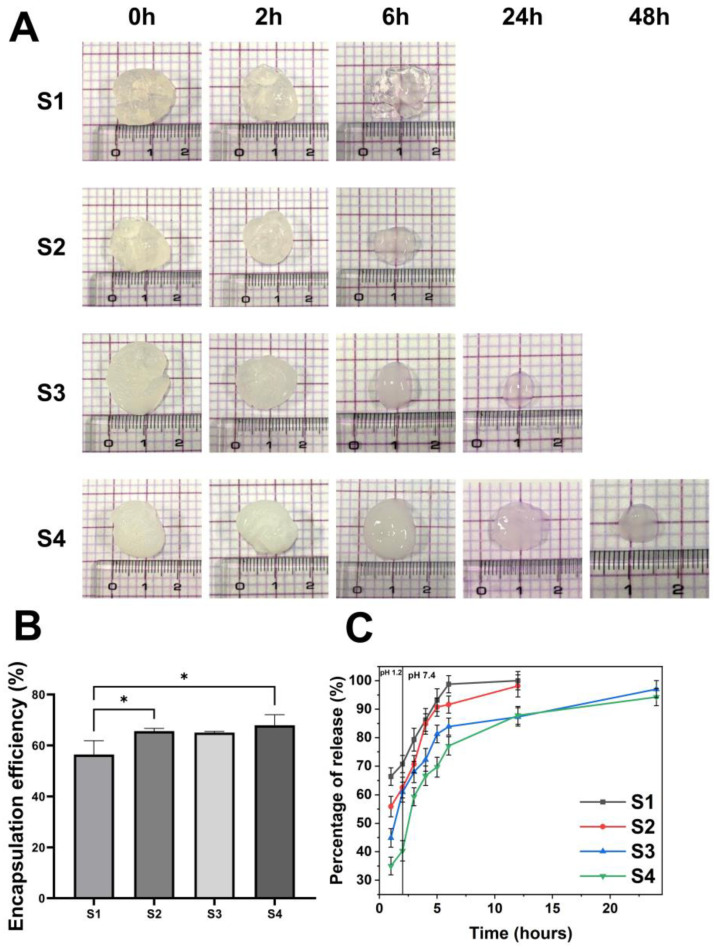
(**A**) Images of Alg-PSI hydrogels with different concentrations of PSI as a function of time: (S1) 0%, (S2) 0.5%, (S3) 1.5%, and (S4) 3% PSI (*w/v*). (**B**) Percentage of AP loaded into Alg-PSI hydrogels with different concentrations of PSI: (S1) 0%, (S2) 0.5%, (S3) 1.5%, and (S4) 3% PSI (*w/v*). The symbol * indicates a significant difference (*p* < 0.05). (**C**) Drug release profile of Alg-PSI hydrogels with different concentrations of PSI at pH 1.2 and 7.4: (S1) 0%, (S2) 0.5%, (S3) 1.5%, and (S4) 3% PSI (*w/v*).

Figure 5B shows the determined loading efficiency of AP in the hydrogel. The loading efficiency of the S1 hydrogel was ~60%, and the drug loss was mostly caused by the porous nature of the alginate network which allowed the drug molecules to diffuse rapidly during the gelation step. On the other hand, the loading efficiency of the S4 hydrogel increased by ~70% with increasing PSI content in the hydrogel structure. This characteristic is comparable to other research that used alginate hydrogels to encapsulate calcium phosphate for boosting medication loading effect [42]. The diffusion ability of the drug was hindered by the presence of PSI in the alginate matrix. As a result, the loss of the model drug during the preparation process was reduced, and ultimately, the loading efficiency was dramatically improved. The drug release rate may be also reduced by such an impact [43].

The degradation of hydrogels is similar to the two basic processes that lead to the degradation of polymer-based materials: surface and bulk degradation [44]. Surface degradation is defined as occurring only at the surface, producing particles of smaller size while retaining the core of the material [45]. Bulk deterioration, on the other hand, causes continuous erosion throughout the material, resulting in fragments as byproducts. Therefore, these two mechanisms can be used together to characterize the degradation mechanism observed in the hydrogel [46] (Figure 5A). Alg is known to undergo proton-catalyzed hydrolysis [47]. Thus, the crosslinked Alg substrate, when exposed to an acidic environment, can be converted to alginic acid, which can lead to a reduced degree of crosslinking and thus faster decomposition. When the hydrogels were exposed to HCl, alginate was hydrolyzed to alginic acid, the COO^−^ group was converted to a bonded carboxylic group, and the electrostatic interactions between the Ca^2+^ and COO^−^ ions almost disappeared. Furthermore, ion exchange can occur between the H+ and free Ca^2+^ ions within the hydrogels [48]. The hydrogels swelled quicker when the solution medium was changed from HCl to phosphate buffer at pH 7.4 [48]. However, it did not attain a high water absorption value because of the loose topology of the hydrogels. Furthermore, ion exchange between H+ ions (produced by carboxylic group ionization in a buffer at pH 7.4) and Na^+^ ions present in the buffer may occur, resulting in carboxylic group absorption. In addition, Alg began to dissolve in the substrate, lowering the weight of the hydrogels and finally fully dissolving. As a result, hydrogels with greater PSI concentrations have a longer hydrogel collapse time, as shown in Figure 5A, because PSI promotes the entanglement of the polymer network. Simultaneously, PSI was not hydrolyzed in the acidic medium, resulting in PSI partially preventing the hydrogel from converting Alg to alginic acid during formulation. As a result, when the hydrogel network was in PBS at pH 7.4, it remained more stable than hydrogels with only Alg substrates. Notably, the hydrogel exhibited symptoms of collapse even at pH 7.4 owing to the alkaline hydrolysis capabilities of PSI. This hydrolysis may persist for up to 24 h in hydrogels with higher PSI values (S3 and S4). The duration for which a material remains in the body is one of the most critical characteristics of drug delivery methods. Their duration should not be too long nor too short to have a therapeutic effect. The duration required to extend the active ingredient and remove the drug delivery material from the body by approximately 24 h is highly reasonable for a medication delivery system for patients with high fever and pain alleviation. These results indicate that S3 and S4 hydrogels have great potential as AP drug delivery systems.

Drug release from the hydrogel was primarily determined by the characteristics of the Alg-PSI matrix and drug encapsulation in the polymer system. The mechanism of drug release from the hydrogel system can be divided into two major pathways: drug release via alginate network breakdown, and drug diffusion via the alginate network [49]. The hydrogels were released differently in media of acidic pH 1.2 and in PBS (pH 7.4), as illustrated in Figure 5C. The AP release of the released hydrogel S1 was greatest at pH 1.2, reaching 70% at 2 h. Drug release via alginate network degradation to alginic acid and hydrogel degradation released the drug from the hydrogel structure. In contrast, in the case of hydrogels S2, S3, and S4, the drug release was somewhat reduced because the presence of PSI inhibited the structural deterioration of the networks, making the hydrogel networks more stable and minimizing the rapid release of AP into the environment. When the medium was changed from pH 1.2 to pH 7.4, the hydrogels had a loose structure due to degradation in the acidic environment, and they could be dissolved in the medium. After 6 h, the S1 and S2 hydrogels begin to dissolve completely. This dissolution took approximately 24 h longer for S3 and over 48 h for S4 due to higher concentrations of PSI in S3 and S4. It is noted that during the first 2 h in the gastric environment, S4 allows significantly less AP release than the other hydrogels and allows for higher release when transported into the intestinal-like environment, improving AP efficacy. Owing to PSI’s ability to be bio-hydrolyzed, it was added to the alginate hydrogel network to extend the time during which AP was released while also assisting in the removal of extra hydrogel from the body.

### 2.4. Cell Viability of Hydrogels

**Figure 6:**
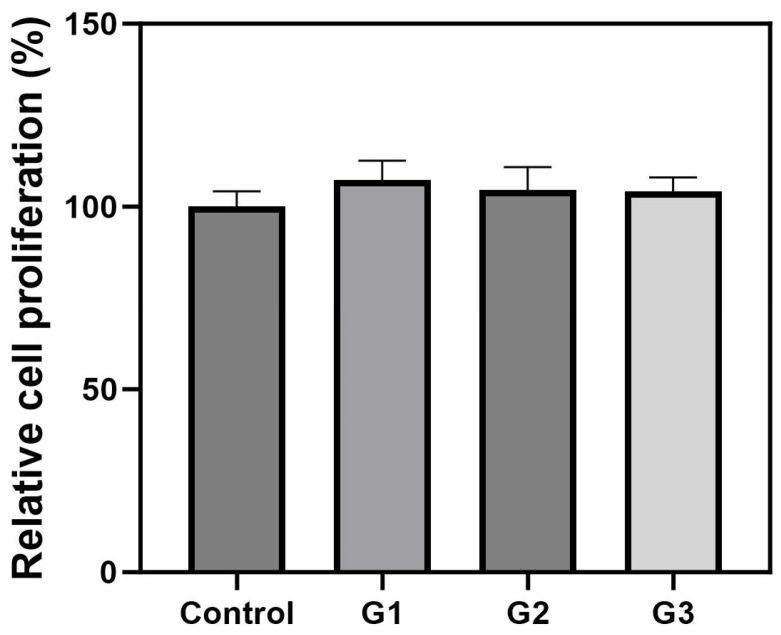
Effects of Alg-PSI hydrogels on the proliferation of NIH/3T3 fibroblasts after 24 h using the 3-(4,5-dimethylthiazol-2-yl)-2,5-diphenyltetrazolium bromide (MTT) assay. (G1) 0.5%, (G2) 1.5%, (G3) 3% PSI (*w*/*v*), and control group. No statistical differences were observed between groups.

An MTT assay was performed to confirm the biocompatibility of Alg-PSI hydrogels at different concentrations. The hydrogels were stabilized for 24 h after gelation, sterilized, and adapted to the cell culture medium. Then, the hydrogels were placed in a hanging insert for indirect interactions with the cells, avoiding direct contact between the hydrogels and cells. The relative cell proliferation (%) was expressed by dividing the absorbance of the treatment groups by the absorbance of the control group. Figure 6 shows slightly higher cell proliferation in the groups treated with Alg-PSI hydrogels than in the control group. These results suggest that the Alg-PSI hydrogels had no cytotoxic effects on cells. In particular, Ca^2+^ ions were used to crosslink the Alg-PSI hydrogels, which altered the cell culture environment but had no adverse effects on the cells. PSI is non-cytotoxic and biocompatible with NIH/3T3 cells; therefore, Alg-PSI hydrogels have excellent cell biocompatibility regardless of the addition ratio [36]. Although not investigated in this study, the number of M-blocks in the alginate structure has been reported to promote cytokine production and affect immunogenicity [50]. These effects can potentially be used to modulate the immunity and control the release behavior of AP and develop new drug delivery systems in the future.

## 3. Conclusions

In this study, we developed an AP analgesic delivery system comprising a PSI-blended alginate hydrogel network. Alginate- and PSI-based hydrogels can be used as long-lasting and biodegradable drug delivery systems. Here, MTT assay revealed that the developed hydrogels were biocompatible. By changing the concentration of PSI, we enhanced the mechanical properties and controlled the biodegradation and release of AP in the hydrogel system. AP was released slowly by the developed system and remained stable for approximately one day. However, to accurately control the encapsulation and release of the drug, the study of detailed physical interactions between PSI and Alg is essential, and further studies through molecular simulations should be performed. This system can reduce the daily intake frequency while maintaining the pain-relieving effect of drugs. Moreover, the hydrogel can be easily removed from the body via PSI bio-hydrolysis in the intestine. Therefore, the developed hydrogel can prolong the analgesic effects of drugs and rapidly eliminate the drug delivery materials from the body to prevent toxicity. Based on the studies mentioned above, additional short-term drug delivery methods can be created and implemented. In the future, it would be possible to further improve the biodegradation and drug release period of Alg-PSI hydrogels, making them suitable for long-term drug delivery systems.

## 4. Materials and Methods

### 4.1. Materials

AP (98.0–102.0%), ASP (≥99%), dimethyl sulfoxide (DMSO, ≥99.9%), hydrochloric acid (HCl, 36.5–38.0%), and methanol (CH_3_OH, ≥99.9%) were purchased from Sigma-Aldrich (Seoul, Republic of Korea). Calcium chloride (CaCl_2_.2H_2_O), Dulbecco’s modified Eagle’s medium (DMEM), penicillin–streptomycin, and trypsin-EDTA solution were purchased from WelGene, Inc. (Daegu, Republic of Korea). NaAlg, sulfolane (99%), mesitylene (98%), phosphoric acid (o-H_3_PO_4_, 85%), fetal bovine serum (FBS), phosphate-buffered saline (PBS, pH 7.4), Dulbecco’s phosphate-buffered saline (DPBS), and 3-(4,5-dimethylthiazol-2-yl)-2,5-diphenyltetrazolium bromide (MTT) were purchased from Thermo Fisher Scientific (Waltham, MA, USA).

### 4.2. Synthesis of Alginate (Alg) Hydrogels

#### 4.2.1. Synthesis of Alg

The alginate hydrogel fabrication method was the same as described above, except for the addition of PSI. Briefly, 2 mL CaCl_2_ solution (1.5%, *w/v*) was added to 2.5 mL NaAlg (1.5%, *w/v*). The gel was stabilized at room temperature for 24 h and washed with distilled water.

#### 4.2.2. Synthesis of Acetaminophen-Loaded Alg (AP/Alg) Hydrogels

Briefly, 0.005 g AP was dissolved in the 2.5 mL NaAlg (1.5%, *w*/*v*). Then, 2 mL CaCl_2_ solution (1.5%, *w*/*v*) was added, and the gel was washed with distilled water after allowing it to stabilize for 24 h at room temperature.

### 4.3. Synthesis of Alg-PSI Hydrogels

#### 4.3.1. Synthesis of PSI

PSI was prepared as previously described [51]. Briefly, 17.5 g ASP and 500 µL o-phosphoric acid (85%) were added to 34 g solvent (mesitylene/sulfolane, 7:3) in a three-necked flask and refluxed under nitrogen pressure. After 4.5 h at 162 °C, the water created in the reaction mixture was removed using a Dean–Stark trap, and the obtained solid was washed with CH_3_OH and water. PSI was dried at 70 °C in an oven. The percentage yield of PSI was over 90%.

#### 4.3.2. Synthesis of Complex Alg-PSI Hydrogels

NaAlg solution (1.5%, *w*/*v*) was prepared in distilled water, and PSI (0.5, 1.5, and 3% *w/v*) was dissolved in DMSO. Then, 2 mL NaAlg solution was added to 0.5 mL PSI solution and stirred for 30 min at 20 °C. After homogenization (NaAlg-PSI), the forming gels were prepared by adding 2 mL calcium chloride (CaCl_2_; 1.5%, *w*/*v*) to 2.5 mL NaAlg-PSI solution. After allowing the gels (Alg-PSI) to stabilize for 24 h at room temperature, they were washed with distilled water and dried at room temperature for subsequent experiments. Different Alg-PSI hydrogels were established using the same steps by immobilizing the NaAlg concentration and varying the PSI concentration (0.5, 1.5, and 3% *w*/*v*).

#### 4.3.3. Synthesis of AP-Loaded Complex Alg-PSI (AP/Alg-PSI) Hydrogels

After preparing NaAlg-PSI solutions, AP (0.005 g) was dissolved in the solution to develop an AP/NaAlg-PSI mixed solution. Then, hydrogels containing the drug were obtained by adding 2 mL CaCl_2_ solution (1.5%, *w*/*v*) to 2.5 mL AP/NaAlg-PSI solution. The gels (AP/Alg-PSI) were stored for 24 h for stabilization, washed with distilled water, and dried at room temperature. The hydrogel samples were sterilized by immersing them in a 70% ethanol solution for 10 min, followed by transfer to DPBS for an additional 1 h. The characterization was performed after the sterilization process.

### 4.4. Characterization

^1^H NMR (Jeol, JNM-ECZR, 500 MHz) was used to characterize PSI. DMSO-D6 was used as the solvent to prepare the ^1^H NMR samples. The number average molecular weight (M_n_), weight average molecular weight (M_w_), and polydispersity index (PDI) of PSI were obtained using GPC (Agilent Technologies 1100). Dimethylformamide was used as the eluent. The Fourier-transform infrared (FT-IR) spectra of Alg, PSI, and Alg-PSI were recorded separately using an FT-IR spectrometer (PerkinElmer Frontier, Shelton, USA) in the range of 4000–700 cm^−1^. X-ray diffraction of PSI, Alg, and Alg-PSI were collected with X-ray diffractometer (Rigaku (SmartLab), Wilmington, MA, USA) with a Cu Kα and produced materials in the 2-theta range from 20° to 80°. Scanning electron microscopy (SEM; Hitachi SU8600, Tokyo, Japan) at the Smart Materials Research Center for IoT (Gachon University, Republic of Korea) was used to examine the morphologies of Alg and Alg-PSI. Prior to scanning, the SEM sample was subjected to a platinum ion coating applied using a sputter coater for 120 s under vacuum.

### 4.5. Rheological Tests of Hydrogels

The stability of the hydrogels was investigated by their dynamic rheological behavior using a rheometer (MCR92, Anton Paar, Graz, Austria) equipped with a parallel-plate geometry and a flat-bottom plate with a diameter of 25 mm. After preparation, the gels of each group were washed with distilled water, and the water was removed. First, the storage (G’) and loss (G″) moduli were measured by a frequency sweep (0.1–10 Hz) under the linear viscoelastic region (LVR) with a fixed strain (0.1%) at 37 °C to confirm the hydrogel. In addition, the viscosity was confirmed according to the shear rate change (0.001–1 s^−1^).

### 4.6. Degradation Analyses of Hydrogels

The degradation rates of the hydrogels were measured in a test medium to correlate the observed drug release with the rate of hydrolysis. The hydrogels were transferred to a water bath in a HCl medium (pH 1.2) and subjected to shaking for 2 h. The hydrogels were then placed in PBS (pH 7.4). All tests were carried out at 37 °C. The surface of the hydrogel was then dried and images of the hydrogel were recorded.

### 4.7. AP Encapsulation Efficiency

AP encapsulation efficiency was determined by extracting the entire drug from the hydrogels. The drug-containing hydrogels were completely dissolved in PBS solution (pH 7.4) by magnetic stirring for 72 h and then completely sonicated to obtain a homogeneous solution (to ensure that the hydrogels had completely degraded and released AP into the PBS solution). The amount of AP was estimated using a UV spectrophotometer at 243 nm and the drug encapsulation efficiency was calculated according to the following equation. The results were obtained from three independent experiments.

$$\%\;\mathrm{Drug}\;\mathrm{encapsulation}=\frac{W_{1}}{W_{2}}\;\times \;100$$

where W_1_ is the actual amount of AP in the hydrogel and W_2_ is the amount of AP theoretically encapsulated.

### 4.8. Acetaminophen Release Tests

The drug release behavior was observed by loading the loaded hydrogels in HCl (pH 1.2) and PBS (pH 7.4) solutions in an orbital shaker bath at 37 °C set at 100 rpm to simulate the digestive environment. At regular intervals, the extracts (1 mL) were removed and immediately replaced with an equal volume of fresh solution. AP release was determined by analyzing the periodic percentage of the extract using UV spectroscopy. The UV-visible absorbance of AP was measured at 243 nm.

### 4.9. In Vitro Cytotoxicity

NIH/3T3 cells were obtained from the Korean Cell Line Bank (Seoul, Republic of Korea). NIH/3T3 fibroblasts were cultured in culture medium (DMEM containing 10% *v*/*v* FBS and 1% *v/v* penicillin–streptomycin) at 37 °C, 5% CO_2_, and 95% air. The biocompatibility of the hydrogels according to the concentration of PSI (0, 0.5, 1.5, and 3% *w*/*v*) was evaluated by MTT assay. Fibroblasts were seeded in 24-well plates at a density of 2 × 10^4^ cells/well and incubated with the culture medium for 24 h. The hydrogel samples were prepared in a size of 25 µL. After 24 h of seeding, the hydrogels were placed on a hanging insert to contact the cells. A sufficient amount of the medium was added to the hanging insert containing the gel to allow it to sink. The control group was not treated with the hydrogel, whereas the other groups (G1, G2, and G3) were treated with hydrogel, respectively. After 24 h, the medium was replaced with culture medium containing 10% *v*/*v* MTT solution (6 mg/mL). After 1 h, the formazan crystals were dissolved in DMSO, and the absorbance was measured using a microplate reader (Agilent Technologies, Santa Clara, CA, USA). The relative cell proliferation (%) was expressed by dividing the absorbance of the treatment groups by the absorbance of the control group.

### 4.10. Statistical Analysis

All data are expressed as mean ± standard deviation. Each experiment was repeated three times, unless otherwise indicated. Statistical evaluation was performed using one-way analysis of variance (ANOVA). A *p* value < 0.05 was considered statistically significant. Statistical analysis was performed using the statistical software GraphPad Prism 9.

## Data Availability

The raw/processed data required to reproduce these findings cannot be shared at this time as the data also form part of an ongoing study.

## Supplementary Materials

The following supporting information can be downloaded at: https://www.mdpi.com/article/10.3390/gels9120980/s1, Figure S1: ^1^H NMR spectroscopy of PSI; Figure S2: GPC of PSI.
