# Supplementary material for: Development of Efficient Sodium Alginate/Polysuccinimide-Based Hydrogels as Biodegradable Acetaminophen Delivery Systems
Source: Gels. 2023 Dec 14;9(12):980. doi: 10.3390/gels9120980 (PMC10743301; doi:10.3390/gels9120980)
Supplement: Supplementary file 1 [file gels-09-00980-s001.zip › gels-2772269-supplementary.pdf]

Supplementary Materials

Development of Efficient Sodium Alginate/Polysuccinimide-Based Hydrogels as Biodegradable Acetaminophen Delivery Systems

Long Toan Trinh [1], Saebin Lim [1], Hyun Jong Lee [1,*] and Il Tae Kim [1,*]

[1] Department of Chemical and Biological Engineering, Gachon University, Seongnam-si 13120, Gyeonggi-do, Republic of Korea

* Correspondence: itkim@gachon.ac.kr (I.T. Kim), hjlee2@gachon.ac.kr (H.J. Lee)

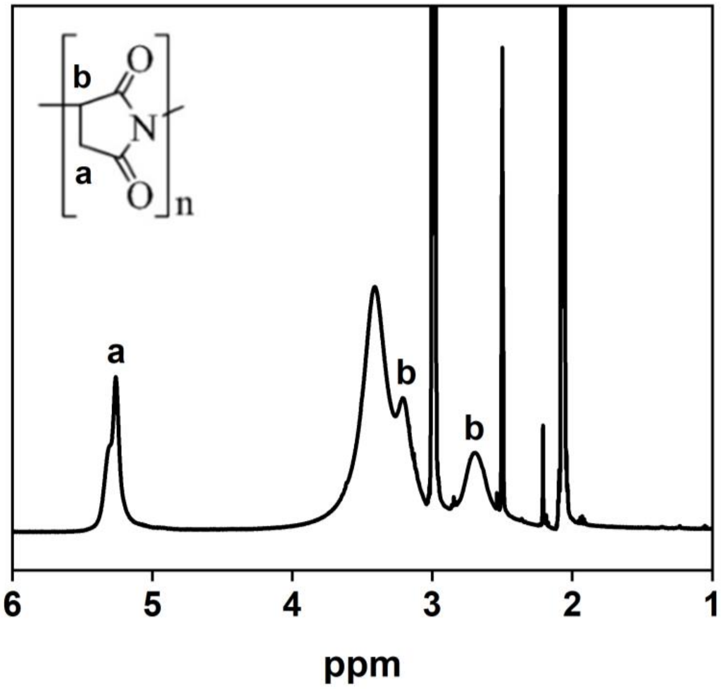

Figure S1. ^{1}H NMR of polysuccinimide.

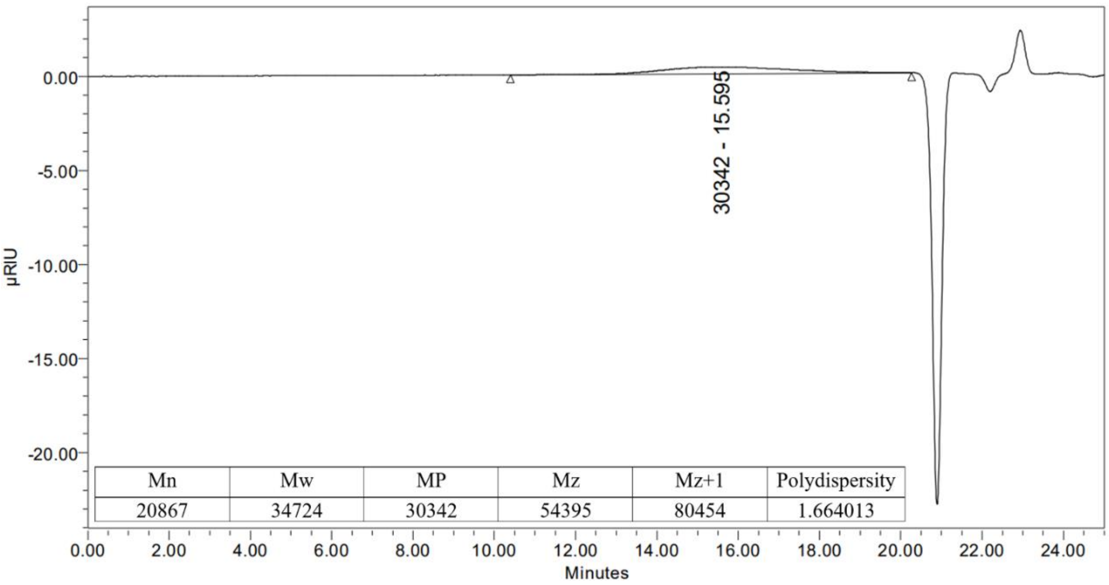

Mn	Mw	MP	Mz	Mz+1	Polydispersity
20867	34724	30342	54395	80454	1.664013

Figure S2. GPC of polysuccinimide.
